# THE INFLUENCE OF DISCRIMINATION ON GAY AND LESBIAN SPOUSAL DEMENTIA CAREGIVERS

**DOI:** 10.1093/geroni/igae098.4317

**Published:** 2024-12-31

**Authors:** Toni Calasanti, Brian de Vries

**Affiliations:** Virginia Tech, Blacksburg, Virginia, United States; San Francisco State University, San Francisco, California, United States

## Abstract

Most research has speculated that discrimination would influence the experiences of gay and lesbian spousal caregivers (e.g., Fredriksen-Goldsen, et al, 2016), but to date little research has revealed the actual impacts of discrimination and where and how it matters. The present study of 13 gay and 16 lesbian spousal dementia caregivers finds that both past experiences and present discrimination influence how they give care. On the one hand, past experiences of discrimination have taught these caregivers to be cautious, even when they have been out for many years and even when they live in what are typically considered to be supportive areas. Further, and even though many respondents reported no accounts of discrimination presently, some reported discriminatory experiences with social service or medical providers; and one gay husband had experienced violence in a care setting. At the same time, and what is less documented, are the ways that our respondents feel that what they learned to do to respond to discrimination prepared them for the challenges of spousal dementia care. This included all that lesbians and gay men faced during the HIV/AIDs epidemic. Having been “othered,” they feel they are more empathetic and sensitive to the stigma that comes with cognitive decline; they have greater self-reliance borne of the struggle with HIV/AIDs or in the face of estrangement from families or broader society; and they rely upon the resources provided by their communities. These results highlight areas both for subsequent novel research and interventions to support same-sex spousal dementia caregivers.

